# Phosphorylation of amyloid beta (Aβ) peptides – A trigger for formation of toxic aggregates in Alzheimer's disease

**DOI:** 10.18632/aging.100362

**Published:** 2011-08-21

**Authors:** Sathish Kumar, Jochen Walter

**Affiliations:** ^1^ Department of Neurology, University of Bonn, 53127 Bonn, Germany

**Keywords:** Alzheimer's disease, amyloid β-peptide, oligomers, phosphorylation, post translational modification, protein folding, conformation and aggregation

## Abstract

Alzheimer's disease (AD) is the most common form of dementia and associated with the progressive accumulation of amyloid β-peptides (Aβ) in form of extracellular amyloid plaques in the human brain. A critical role of Aβ in the pathogenesis of AD is strongly supported by gene mutations that cause early-onset familial forms of the disease. Such mutations have been identified in the APP gene itself and in presenilin 1 and 2. Importantly, all the identified mutations commonly lead to early deposition of extracellular plaques likely by increasing the generation and/or aggregation of Aβ. However, such mutations are very rare and molecular mechanisms that might trigger aggregation and deposition of Aβ, in the most common late onset AD are largely unknown. We recently demonstrated that extracellular Aβ undergoes phosphorylation by a cell surface-localized or secreted form of protein kinase A. The phosphorylation of serine residue 8 promotes aggregation by stabilization of β-sheet conformation of Aβ and increased formation of oligomeric Aβ aggregates that represent nuclei for fibrillization. Phosphorylated Aβ was detected in the brains of transgenic mice and human AD brains and showed increased toxicity in Drosophila models as compared with non-phosphorylated Aβ. Together, these findings demonstrate a novel molecular mechanism that triggers aggregation and toxicity of Aβ. Thus, phosphorylation of Aβ could be relevant in the pathogenesis of late onset AD. The identification of extracellular protein kinase A should also stimulate pharmacological approaches to decrease Aβ phosphorylation in the therapy and/or prevention of AD.

## INTRODUCTION

Alzheimer's disease (AD) is the most common form of dementia in the ageing population and affects millions of people worldwide [1]. At the neuropathological level, AD is characterized by neuronal cell loss and the combined presence of two lesions in the brain - extracellular amyloid-beta (Aβ) plaques and intracellular neurofibrillary tangles (NFTs) [2]. The extracellular deposits contain aggregated Aβ peptides [3], while intraneuronal tangles are aggregates of hyper-phosphorylated forms of the neurofilament-associated protein tau [4]. Evidence suggests that the pathogenesis of AD involves deleterious neurotoxic effects of both types of aggregates [5;6]. However, numerous studies strongly support a critical role of Aβ aggregates in the initiation phase of AD pathogenesis, while tau might mediate toxicity and impairment of neuronal function [5-9].

Aβ is a proteolytically processed fragment of the amyloid precursor protein (APP) [10;11]. It occurs in different length variants with peptides of 40 amino acid residues (Aβ40) and 42 amino acid residues (Aβ42) being the most prevalent. The longer Aβ42 variant has a much higher propensity to form aggregates. Genetic studies identified mutations in three genes that cause familial forms of AD (FAD): APP, presenilin-1 (PS1), and presenilin-2 (PS2) [12]. Mutations in each of these genes result in elevated levels of Aβ production and/or promote its aggregation. This genetic correlation strongly favours the key role of Aβ in AD. However, mutations in APP and PS are very rare, and the causes of the much more common late onset forms of AD (LOAD) are largely unidentified. In line with a significant role of Aβ in pathogenesis, recent data show that various post-translational modifications of Aβ promote its aggregation and therefore could play important roles in the initiation of LOAD.

### Generation of Aβ by proteolytic processing of APP and effects of AD associated mutations

APP is a type I membrane protein and ubiquitously expressed in most cell types. Alternative mRNA splicing leads to several cell type and development-specific isoforms [2]. In addition, two homologous APP-like proteins (APLPs) have also been identified, that together form a small protein family with important physiological functions in perinatal and postnatal development and cell communication [13]. However, APLPs do not contain the Aβ sequence and thus APP is the sole source of Aβ peptides in the brain [14].

Aβ is produced during normal cellular metabolism and secreted to the extracellular milieu of the human brain and also found in cerebrospinal fluid (CSF) [15;16]. The presence of Aβ in the CSF of nondemented individuals and in the media from neuronal cell cultures during normal metabolism could indicate a physiological role of Aβ in the central nervous system [17]. Suggested physiological function of Aβ includes ion channel modulation [18], kinase activation [19], regulation of cholesterol transport [20], protection against metal-induced oxidative damage [21], learning and memory [22] and transcriptional regulation of AD-associated genes [23].

**Figure 1:**
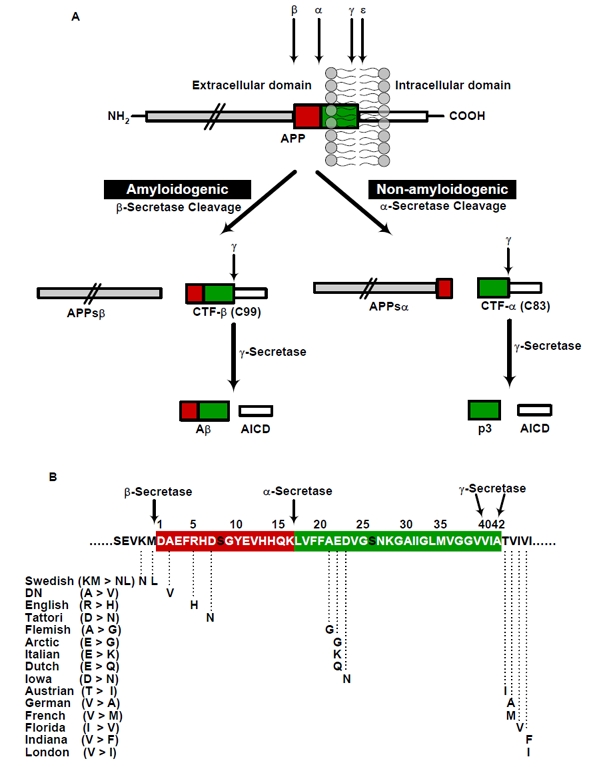
Schematic representation of generation of Aβ by proteolytic processing of APP and the familial AD causing APP mutations **(A)** Two pathways (β/γ and α/γ) of APP proteolysis. APP can be cleaved by either β- or α-secretase, which is then followed by γ-secretase cleavage results in the generation of either the p3-fragment **(non-amyloidogenic)** or an Aβ **(amyloigenic pathway)**. The designation of secretases, substrates and products are depicted, **(B)** Representation of APP familial AD causing mutations that are identified around N- and C-terminal and in the middle region of Aβ. The amino acid residues are numbered according to Aβ sequence. The swedish mutation (KM>NL) at N-terminus of Aβ̣ near to β-secretase cleavage site increases the total production of Aβ, whereas the mutations C-terminus of Aβ results in increased production of Aβ42 by altering γ-secretase activity. The mutations in the middle region of Aβ might decrease the α-secretory cleavage, facilitate the amyloidogenic processing, promote the Aβ production and/or increases the propensity of Aβ aggregation or stabilizes the Aβ against clearance by different proteases.

The generation of Aβ is initially starts with a cleavage of APP by β-secretase at the N-terminus of the Aβ domain (Figure 1A). This cleavage results in the shedding of the APP ectodomain and the generation of a membrane bound carboxyl (C)-terminal fragment (CTF-β). Subsequently, γ-secretase mediates the apparently intramembranous cleavage of CTF-β resulting in the liberation of Aβ into conditioned media of cultured cells or extracellular fluids of the brain or the periphery [2;11]. Alternatively, APP can also be cleaved in a non-amyloidogenic pathway that involves initial cleavage by α-secretase within the Aβ domain thereby precluding the subsequent generation of Aβ peptides (Figure 1A) [24].

The mutations within APP that causes early onset AD (EOAD), are all located within or close to the Aβ domain. Notably, a double mutation in APP at the cleavage site for β-secretase that cause EOAD increases the β-secretory cleavage resulting in an overall higher production of Aβ peptides (see Swedish mutation, Figure 1B) [25]. Additional EOAD-associated mutations located close to the cleavage site for γ-secretase at c-terminal of Aβ also alter the proteolytic processing of APP (Figure 1B). These mutations increase the ratio of Aβ42/40 peptides thereby promoting the relative production of Aβ variants with higher propensity to aggregate [26]. Mutations found in the middle of the Aβ domain might exert different effects (Figure 1B), (1) they might decrease the α-secretory cleavage of APP thereby facilitating amyloidogenic processing of APP [27], (2) these mutations could also increase the aggregation [28], (3) and/or alter the degradation by different proteases [29].

Beside the APP gene, two additional genes have been identified to contain mutations that lead to EOAD [30]. Both genes encode highly homologous PS proteins that are critical components of the γ-secretase complex, which includes three additional proteins such as nicastrin, APH-1 (anterior pharynx-defective 1), and PEN-2 (presenilin enhancer 2) to exert γ-secretase activity in cells [31]. The mutations in PS1 or PS2 also alter γ-secretase activity and/or cleavage specificity, resulting in higher ratios of Aβ42/40 [31]. Together, all mutations in the three genes known to be associated with EOAD affect the generation and/or aggregation of Aβ [25;27]. However, as mentioned before such mutations are very rare and mechanisms that increase the aggregation and accumulation of Aβ and cause the much more common sporadic forms of AD (>95% of all cases), are largely unknown. According to the ‘amyloid hypothesis‘, accumulation of Aβ in the brain is the primary influence driving AD pathogenesis. The rest of the pathogenic events, including impaired synaptic function and cell communication [7;32;33], activation of microglia and astrocytes [34;35], neuronal ionic homeostasis and oxidative injury [36], mitochondrial dysfunction [37], altered kinase/phosphatases activities leading to formation of neurofibrillary tangles containing tau protein, is proposed to result from an imbalance between Aβ production and Aβ clearance [38].

### Aβ aggregation – routes to neurotoxic assemblies

**Figure 2:**
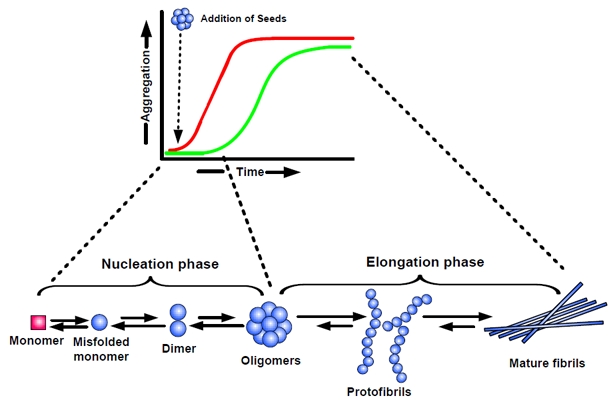
Nucleation-dependent polymerization model of amyloid aggregation Amyloid formation consists of two phases: (i) a nucleation phase/lag phase, in which monomers undergo conformational change/misfolding and associate to form oligomeric nuclei, and (ii) a elongation phase/growth phase, in which the nuclei rapidly grow by further addition of monomers and form larger polymers/fibrils until saturation. The ‘nucleation phase‘, is thermodynamically unfavourable and occurs gradually, whereas ‘elongation phase’, is much more favourable process and proceeds quickly. Thus, kinetics of amyloid formation is well represented by a sigmoidal curve with a lag phase followed by rapid growth phase (green curve). The rate limiting step in the process is the formation of nuclei/seeds to promote aggregation. Thus, amyloid formation can be substantially speedup by the addition of preformed seeds (nuclei). The addition of seeds reduces the lag time and induces faster aggregate formation (red curve).

Amyloid formation in AD is conceptualized as a complex process of protein aggregation, involving the misfolding of Aβ into soluble and insoluble assemblies [39]. Monomeric Aβ is mainly composed of α-helical and/or unordered structure, whereas the misfolded polymers are rich in β-sheet conformation. The conformational changes leading to the formation of extended β-sheets promotes homophilic interactions and eventually leads to Aβ oligomer formation. Kinetic studies have suggested that misfolding of monomeric Aβ precedes formation of oligomers, which then serve as seeds/nuclei for accelerated fibril growth (Figure 2) [40].

A widely accepted concept for the formation of amyloid fibrils is the nucleation-dependent polymerization model [41-43], which separates the fibrillization process into a nucleation phase and an elongation phase. Nucleation requires the self-association of soluble monomers, which is thermodynamically unfavourable and so occurs slowly. In the nucleation phase, monomers undergo conformational changes and self-associate to form oligomeric nuclei that are rich in β-sheets. Once the nucleus is formed, assembly of larger aggregates and fibril elongation, a much more favourable process and proceeds rapidly. As a result, the kinetics of amyloid fibril formation is well represented by a sigmoidal shape with a nucleation phase/lag phase followed by a rapid growth phase, followed by a saturation phase (Figure 2; green curve). The lag phase is determined by the critical concentration of nuclei, which represent seeds for further growth of the polymers finally resulting in mature fibrils. Accordingly, the lag phase of aggregation can be shortened by addition of preformed seeds (Figure 2; red curve).

In a landmark discovery, Pike et al., [44], established that innocuous monomers of Aβ become neurotoxic upon aggregation. It was further shown that toxicity of Aβ involved self-association of monomers into oligomers and higher aggregated forms [45]. This is further supported by *in vitro* [46-48], and *in vivo* studies showing that oligomeric and pre-fibrillar Aβ assemblies are potent neurotoxins [5;49;50]. A correlation between soluble oligomeric Aβ levels and the extent of synaptic loss and severity of cognitive impairment further corroborate the findings [7;32]. Thus, neurotoxicity appears to require toxic oligomeric assemblies of Aβ. The formation of such neurotoxic assemblies in the brain generated due to higher production and/or decreased clearance of Aβ [51;52].

### Effect of post-translational modification on aggregates formation, toxicity and clearance

Amyloid plaques in the human AD brain are known to contain a heterogeneous mixture of Aβ peptides [53]. In addition to main Aβ species (Aβ40 and Aβ42), a variety of post-translationally modified variants have been identified [54], including truncation [55-58], racemization [59;60], isomerization [61;62], pyroglutamination [63;64], metal induced oxidation [65] and phosphorylation [66-68].

The N-terminal truncated variants of Aβ beginning at amino acid 3, 11 and 25 are present in senile plaques and vascular amyloid deposits [56;57;57;69-71]. The truncated Aβ25-35 is shown to favour aggregation *in vitro* [72]. Due to potential toxic effects of truncated Aβ25-35, it has been frequently used for aggregation or toxicity studies [73]. Racemization of Aβ at Asp7, Asp23 and Ser26 was reported in the human brain and aggregation properties of Aβ were influenced by the position of the racemized residue [59;60]. Isomerization of aspartate residues at position 1, 7 and 23 of Aβ results in structural transition of Aβ and also shown to occur *in vivo* [62]. Isomerization of Aβ promotes fibril formation *in vitro* and resistance to proteolytic degradation [61]. In addition Aβ can undergo pyroglutamination also resulting in faster aggregation [74;75].

Thus, post-translational modifications of Aβ could promote oligomer and aggregate formation, thereby also reducing the degradation by a variety of proteases [76-79]. Modified Aβ peptides show enhanced cytotoxicity as compared to non-modified peptides [73], and serve as seeding species for Aβ aggregate formation *in vivo* [66;74;78]. These post-translationally modified Aβ variants appear to be present at an early stages of the disease [58;66;71;74].

### Extracellular phosphorylation

Phosphorylation is an important reversible post-translational modification that regulates the structural and functional properties of proteins in health and disease [80]. Phosphorylation is a key step in the regulation of protein activity, cell cycle control, gene regulation, learning and memory [81]. In addition to intracellular protein kinases (PKs), extracellular PK activities have also been described [82]. These extracellular kinases phosphorylate cell-surface proteins and soluble extracellular substrates, and thus could affect many physiological processes involving cell-cell contacts, cellular differentiation and proliferation, ion transport [82]. Depending on the localization, these PKs are differentiated as ecto-PKs and exo-PKs. Ecto-PKs are localized at the external surface of the plasma membrane (membrane bound) where they exert their catalytic activity [83-86]. Exo-PKs are secreted/shedded to the extracellular milieu [87;88]. Ecto- and Exo-PKs can phosphorylate extracellular membrane bound proteins and soluble proteins Both Ecto- and Exo-PKs use extracellular ATP as co-substrate, which can be released by intact cells [89;90]. Extracellular ATP plays physiological roles in neurite outgrowth, neurotransmission and glial communication [91]. The release of extracellular ATP is mediated by metabotropic (P2Y) and ionotropic (P2X) receptors, both are widely expressed in the nervous system [92]. In the brain, extracellular ATP is present in low nanomolar concentrations. However, the local ATP concentration can increase upon certain stimuli, including synaptic activation [89;93], inflammation [94] and ischaemia *in vivo* [95]. Therefore, extracellular phosphorylation is likely to play a role in normal as well as pathological processes in the brain.

### Phosphorylation of Aβ

A variety of AD associated proteins including APP [96-98], BACE [99;100], PS [101;102] and tau [103;104], are shown to be phosphorylated. Phosphorylation of these proteins affects subcellular trafficking, interaction with adapter proteins, signal transduction cascades, APP processing, Aβ generation and tangle formation. In AD, tau is shown to be abnormally hyperphosphorylated at several Ser/Thr residues. Hyperphosphorylation and subsequent accumulation of neurofilament subunits is a typical feature of the AD brain [105;106]. However, the pathophysiological relevance of tau phosphorylation is still under debate.

*In silico* analysis revealed that Aβ contain potential phosphorylation sites at serine residue at 8^th^ and 26^th^ position and tyrosine residue at 10^th^ position. Aβ can undergo phosphorylation by protein kinase A and cdc 2 *in vitro* [68], as well as by cultured cells and in human CSF (Kumar, 2009; URN: urn:nbn:de:hbz:5N-18193).

We recently showed that Aβ is phosphorylated at serine-8 by extracellular protein kinase A. Phosphorylation of Aβ promoted the formation of toxic aggregates [66]. The formation of small soluble oligomers is associated with the conformational transition of Aβ from α-helical and random coiled state to a β-sheet structure, as demonstrated by circular dichroism. Phosphorylation-state specific antibodies were used in western-blotting and immunohistochemistry to demonstrate the occurrence of phosphorylated Aβ in murine AD models and AD patient's brain tissue. Notably, these antibodies further confirmed that phosphorylation occurs at free extracellular Aβ rather than at the full-length APP or β-CTF, the precursors of Aβ peptide. Phosphorylated Aβ co-localized with non-phosphorylated Aβ in extracellular plaques [66]. Interestingly, phosphorylated Aβ appeared to be concentrated in the centre of individual plaques and was detected as early as at 2 months of age in APP transgenic mice, and then accumulated with aging. The detection of phosphorylated Aβ in oligomeric assemblies in mouse brain homogenates suggested that phosphorylation also increases aggregation of Aβ *in vivo*. Therefore, we hypothesize that phosphorylation of Aβ might act as a conformational switch, thereby promoting the formation of aggregates.

To test the effect of Aβ phosphorylation on toxicity *in vivo*, transgenic Drosophila models were employed. Since Drosophila allows the selective expression of Aβ independent of its precursor APP [107;108], transgenic Drosophila flies expressing either the wild type Aβ (AβWT) or pseudophosphorylated mutant (AβS8D) were generated. When expressing AβWT and AβS8D mutant in photoreceptor cells in Drosophila eyes, the pseudophosphorylated AβS8D variant showed significant cell degeneration compared to AβWT, demonstrating increased toxicity of pseudophosphorylated Aβ. Notably, pseudophosphorylated AβS8D also accumulated to much higher levels in aged flies than AβWT, strongly indicating increased aggregation. In addition, transgene expression in the fly brain showed stronger age-dependent accumulation of pseudophoshporylated Aβ peptides as compared to AβWT. The increased toxicity of pseudophosphorylated Aβ was revealed by altered climbing behaviour upon aging. This progressive age-dependent phenotype, correlates with Aβ peptide accumulation, indicating that pseudophosphorylated Aβ can mimic the effect of phosphorylation on Aβ aggregation *in vivo* [66].

The Aβ plaque formation could be induced by inoculation of amyloid containing brain homogenates from human or transgenic mouse into brains of monkeys or APP transgenic mice, suggesting the occurrence of nucleation-dependent fibrillization *in vivo* [109;110]. As phosphorylation of Aβ promotes oligomer formation, phosphorylated Aβ oligomers could serve as seeds or nuclei that increase the rate of aggregation. In agreement with this hypothesis, the nuclei of phosphorylated Aβ were capable to promote aggregation of non-phosphorylated Aβ *in vitro* [66].

Several proteases or peptidases have been reported that are able to cleave Aβ and thereby contribute to efficient removal of Aβ in the brain [52;111]. It will therefore also be interesting to assess the effect of phosphorylation on protease dependent degradation of Aβ.

## CONCLUSION

Increasing evidence suggests that phosphorylation of proteins involved in several neurodegenerative diseases and plays a serious role during the pathogenesis [67;112;113]. The role of phosphorylation in modulating the aggregation and fibrillogenesis of tau in AD and α-synuclein in Parkinson's disease (PD) is currently a subject of intense investigation [103;114;115]. Our studies provide evidence that Aβ can undergo phosphorylation. Phosphorylation promotes conformational transition and formation of toxic aggregates. Further, phosphorylated Aβ aggregates could serve as endogenous seeds triggering further aggregation of soluble, extracellular Aβ into plaques in the brain. Phosphorylation stabilizes the Aβ against degradation by various proteases *in vitro* and in cell cultures (Kumar et al., Unpublished data). The stabilization of Aβ by phosphorylation might play a crucial role in AD pathogenesis, because it would eventually result in increased concentrations of this peptide in the brain. Therefore, inhibition of extracellular kinases or stimulation of Aβ dephosphorylation could be pursued as valuable targets to prevent or slow down the progression of AD. Further, the detection of phosphorylated Aβ in biological fluids could also be explored for evaluation as biomarkers. Together, phosphorylation of Aβ might have very important implications for AD pathogenesis and offer novel therapeutic avenues.
